# Countermovement Jumps in Pre-School Children Aged 3 to 6 Years: How Much Can Arm Swing Help in Performance?

**DOI:** 10.3390/sports13110387

**Published:** 2025-11-04

**Authors:** Vilko Petrić, Jera Gregorc, Sanja Ljubičić

**Affiliations:** 1 Faculty of Teacher Education, University of Rijeka, 51000 Rijeka, Croatia; vilko.petric@ufri.uniri.hr; 2 Faculty of Education, University of Ljubljana, 1000 Ljubljana, Slovenia; jera.gregorc@pef.uni-lj.si

**Keywords:** biomechanics, coordination, countermovement jump, motor development, preschool children, reactive strength index, vertical jump

## Abstract

Vertical jumping is a fundamental motor skill that develops rapidly in early childhood, yet the biomechanical contribution of arm swing in preschool-aged children remains unclear. This study aimed to investigate how arm swing influences countermovement jump (CMJ) performance in typically developing children aged 3 to 6 years. A total of 411 children (53.5% girls; mean age: 4.9 ± 1.1 years) from four European cities participated in this cross-sectional study. Each child completed five CMJs with and without arm swing using the Optojump system, measuring variables such as jump height, flight time, contact time, power, the reactive strength index (RSI), pace, and verticality. The results revealed a significant increase in jump height when using arm swing for both boys (+15%) and girls (+12.5%) (*p* < 0.001), yet power output, the RSI, pace, and verticality decreased significantly (*p* < 0.05). These findings suggest that preschool children are not yet biomechanically efficient in integrating arm movements into vertical jumping due to immature neuromuscular coordination. Although arm swing improves jump height, it does not enhance overall movement efficiency at this developmental stage. This study highlights the need for multidimensional and age-appropriate assessment protocols to better understand motor integration during early childhood.

## 1. Introduction

Preschool age represents an extremely important developmental period during which children acquire fundamental motor skills such as running, jumping, and catching, which form the foundation for further motor learning [1]. High-quality performance of these basic movements from an early age is associated with a greater likelihood of maintaining or improving motor abilities throughout childhood and adolescence [2]. However, during this period, atypical movement patterns are often observed, which can be attributed to immaturity of motor skills, underdeveloped sensory integration, and limited neuromuscular control [3]. These deficits are particularly noticeable in the execution of locomotor tasks, including jumping, where children often fail to optimally coordinate arm and leg movements, resulting in reduced movement efficiency [4].

Jumping is one of the most important fundamental motor skills that children begin to acquire as early as the second year of life, and by the end of the preschool period, they should typically be proficient in using various forms of jumps [5]. Proper execution of jumps requires full-body coordination and the integration of multiple motor abilities—strength, speed, balance, and precise movement control [6]. The vertical jump, in particular, is valuable for evaluating lower limb muscle strength and overall motor readiness [7], and due to its biomechanical complexity, it is also considered an indicator of more advanced motor skills [8].

The vertical jump is commonly performed in two basic variations—with and without an arm swing. A jump without arm swing isolates the work of the lower limbs and reflects pure muscular strength, while a jump with arm swing involves the entire body and utilizes biomechanical and coordination advantages to achieve greater jump height [9,10]. The role of arm swing in jump biomechanics has been confirmed by numerous studies, showing that such jumps result in significantly higher values of peak ground reaction force, the rate of force development, and force impulse, especially in the vertical plane [11,12]. These differences are further emphasized in unilateral jumps, where arm swing significantly contributes to the mechanical outcome of the movement.

Despite numerous studies in the sports context, existing knowledge about vertical jumps in preschool children remains limited. Most normative data are based on samples of school-aged children [13,14], while significantly fewer studies have been conducted on samples of preschool-aged children [7,15,16]. The results of these studies [7,15,16], as well as additional research [17,18], indicate considerable intrasubjective variability, likely due to neurodevelopmental discrepancies between the perceptual and motor systems at this age. Furthermore, most previous studies have focused exclusively on jump height, neglecting other important biomechanical parameters such as force, force impulse, and the reactive strength index (RSI) [5,7].

Additionally, little is known about interindividual and bilateral differences in jump performance among preschool children, as well as their ability to utilize arm swing to enhance performance [19]. Research on vertical jumps with and without arm swing in children enables a deeper understanding of their motor abilities [5]. Differentiating between these two types of jumps provides information not only about strength but also about the level of whole-body movement integration—which is crucial for developmental, diagnostic, and training purposes [16].

Given the aforementioned insights and gaps in the literature, the aim of this research is to examine the biomechanical characteristics of jumps in preschool children, with a focus on the difference between jumps with and without arm swing, and to more precisely clarify the biomechanical mechanisms by which arm swing contributes to jump efficiency in children aged 3 to 6 years.

## 2. Materials and Methods

### 2.1. Study Participants

A convenient sample of 411 pre-school boys and girls aged 3–6 years old from four European cities, Rijeka (Croatia), Zagreb (Croatia), Ljubljana (Slovenia) and Koper (Slovenia), were recruited in this observational, cross-sectional study ( mean (SD): [age = 4.9 (1.1) years, height = 111.2 (9.3) cm, weight = 20.0 (4.2) kg, 53.5% girls]). The inclusion criteria involved children aged 3–6 years with typical development and without any locomotor or mental disorders and diseases who were enrolled in daycare. The analysis was conducted with the following parameters using a G*power calculator and the *F* test with the analysis of covariance (ANCOVA): 2 groups (boys vs. girls), 1 covariate (age), a statistical power of 0.80, and a a priori α level of <0.05, a total sample size of *n* = 411, and a pre-requested denominator of 10. The expected effect size (ES) was f = 0.20, indicating that differences between the groups of small to medium ES could be detected. The participants were recruited from one kindergarten representing one of each European city (Zagreb, Rijeka, Ljubljana, and Koper). In each kindergarten, approximately 200 children were in the daycare, of which we wanted to collect at least 50% of the sample. Of the 800 children, we were able to collect the data from *n* = 411 boys and girls aged 3–6 years old. The ES calculation based on the obtained sample size can be seen in this section. According to data analysis standards, we assured similar representation of boys and girls within each age group to omit the possibility of an error. Before entering the study, parents or guardians were instructed about the main aims and hypotheses of the study, as well as the dissemination of the findings and potential benefits for their children. Each child’s parents/guardians provided informed consent before data collection. The research related to human use was performed with all the relevant national regulations and institutional policies, in accordance with the tenets of the Helsinki Declaration (2013), and was approved by the Faculty of Teacher Education, University of Rijeka.

### 2.2. Jumping Performance

The vertical jump test was evaluated using the Optojump photocell system (Microgate, Bolzano, Italy), a reliable and valid infrared platform consisting of two parallel bars (receiver and transmitter units) [20]. Each bar was placed 1 m apart in a parallel position and was connected to a personal computer with software to quantify jump height [20]. The flight time of vertical jumps was measured with an accuracy of 1/1000 s, and the estimated jump height was calculated by the following formula: 9.81 × flight time^2^/8 [21]. To determine jumping outcomes, we selected two tests: countermovement jumps (CMJs) without arm swing and CMJs with arm swing [20]. From a kinematic perspective, children were instructed to start both tests from the standing position with the trunk and knees fully extended and the feet shoulder-width apart; then they executed a fast downward movement with approximately 90° knee flexion and a fast upward movement to jump as high as possible; this was performed by keeping the hands on the hips throughout the whole movement (a CMJ without arm swing) or by swinging back with the arms during the downward movement and forward during the upright movement (a CMJ with arm swing) [22]. Each test was performed 5 times with a 5 min rest period between each trial. The CMJ tests have been extensively used to assess vertical performance and power output, and their reliability, validity, and utility properties have been confirmed in children [22]. The Optojump software (version 1.13.24.0) automatically generated data regarding contact time (s), flight time (s), jump height (cm), power (W/kg), pace (steps/s), and verticality [23], excluding the RSI, which was calculated by dividing jump height by the ground contact time.

### 2.3. Data Analysis

Data normality was calculated using the Kolmogorov–Smirnov (K-S) test. Basic descriptive statistics are presented as the mean (standard deviation; SD) or as the median and interquartile range (25th and 75th percentiles) for normally and not-normally distributed variables. We examined the differences for normally distributed data with Student’s *t*-test, and for not-normally distributed data, the Mann–Whitney U test was performed. Changes between CMJs with and without arm swing were calculated as follows: ∆ (%) = ((CMJ—arm swing) − (CMJ—without arm swing))/(CMJ—without arm swing) × 100. The magnitude of the change between the two measurements (CMJs with and without arm swing) was examined with the following effect size (ES), which was proposed by Hopkins et al. [24]: trivial (ES < 0.2), small (0.2 < ES < 0.5), moderate (0.5 < ES < 0.8), large (0.8 < ES < 1.6), and very large (ES > 1.6). To examine sex- and age-specific changes in CMJs with and without the arm swing, we used repeated measures ANOVA (RMANOVA) with the factor ‘time’ (CMJ and CMJA) and sex and age factors entered in the ‘between subjects differences’ column and found no significant main effects for the interaction between changes in a certain CMJ outcome variable and ‘sex’ (*p* = 0.235–0.766), ‘age’ (*p* = 0.113–0.877), and ‘time × sex × age’ (*p* = 0.336–0.977). All analyses were performed in Statistical Packages for Social Sciences ver. 26 (SPSS Inc., Chicago, IL, USA). The significance was set at *p* < 0.05.

## 3. Results

The use of arm swing significantly influenced CMJ performance in preschool children. In both boys and girls, performing the CMJ with arm swing resulted in increased contact time, flight time, and jump height, whereas pace, the reactive strength index (RSI), and verticality tended to decrease. Power output showed minimal changes in boys but a slight decrease in girls (Table 1). These patterns indicate that arm swing contributes primarily to vertical displacement while slightly compromising reactive and pacing characteristics.

## 4. Discussion

In this chapter, we first briefly present the key findings of our research, followed by an interpretation through the synthesis of these findings in the context of developmental biomechanics. The children in our study were between the ages of 3 and 6, which represents a period of intense reorganization of motor patterns [25,26]. At this developmental stage, the coordination of sensorimotor systems is still undergoing differentiation [27], and therefore, children are not yet capable of complete biomechanical optimization of motor tasks such as vertical jumping.

The primary objective of this article was to examine the influence of arm swing on vertical jump performance in preschool children and to identify specific biomechanical adaptation differences between boys and girls. According to developmental principles, it is expected that arm swing at this age is not yet fully integrated into the overall motor pattern of the jump. This may result in an incomplete vertical trajectory, inefficient muscle group synergy, and suboptimal timing of the take-off phase [28].

Based on the results obtained, we highlighted the main biomechanical characteristics distinguishing jumps with (CMJA) and without (CMJ) arm swing and discussed possible developmental factors that may contribute to these differences. On this basis, we also propose directions for further research aimed at gaining a deeper understanding of the mechanisms involved in the development of vertical jumping in early childhood.

The first finding relates to the effect of arm swing on jump height. This finding aligns with the study by Gillen et al. [29], which found that children (average age: 12.1 ± 1.1 years) jumped 15–17% higher when using arm swing. Koren et al. [24] examined differences between countermovement jumps (CMJs) and countermovement jumps with arm swing (CMJA) in a sample of 79 children aged 4 to 6 years. The effect of arm swing was found to increase with age, with the greatest difference (+1.5 cm) observed in the oldest group (6-year-olds); CMJA performance was 11% higher in boys and 10.5% in girls [24]. The increase in jump height with age was attributed to developmental progress in the CMJ technique. Coordination between the upper and lower body appears to play an important role in CMJ performance, as evidenced by the progressively greater improvements in CMJA compared to CMJs [24].

We believe that the effect of arm swing is best initially explained from a biomechanical perspective. During the eccentric phase, arm swing increases the velocity of the upper body, resulting in increased kinetic energy and transfer of mechanical energy to the lower limbs in the concentric phase [29,30]. An unexpected biomechanical observation emerged: although an increase in power, reactive strength index (RSI), and pace was anticipated, the results did not confirm these expected improvements. Despite achieving higher jump heights in CMJA compared to CMJs, other performance indicators remained unchanged or declined. For example, the RSI—representing the ratio between jump height and ground contact time and frequently used as an indicator of eccentric–concentric efficiency of the muscle–tendon unit [31,32]—slightly decreased in both boys and girls, suggesting a less efficient use of elastic energy.

Another measured parameter that does not support the biomechanical efficiency of CMJA over CMJs is movement pace, which refers to the timing of movement from initiation to take-off and indirectly reflects neuromuscular organization. A shorter time to take-off usually indicates greater explosiveness and better timing of muscle activation [33]. However, in our study, the take-off time in CMJA actually increased on average (boys: +0.06 s; girls: +0.04 s), suggesting that arm swing in preschool children is not yet fully coordinated within the motor pattern and may even negatively impact timing efficiency.

We also expected that the higher jump might be due to greater take-off power, but our data did not confirm this either.

Synthesis of these findings allows for a possible explanation in light of vertical movement orientation. Verticality is defined as the ratio of vertical to horizontal velocity components and reflects the directionality of force application. Higher verticality typically indicates a more efficient biomechanical strategy and is associated with better intersegmental coordination [4,34]. In our study, however, verticality decreased with arm swing, suggesting that the jump was not purely upward but involved more off-axis or dispersed force directions—possibly indicating a forward jump component. This finding highlights the importance of multidimensional jump analysis in preschool children, such as simultaneous 3D motion capture.

Eythorsdottir et al. [34] also emphasize that caution is needed when interpreting jump height, as it is calculated via formulas. The longer flight time observed in our participants may be attributed not to increased vertical lift but rather to greater leg flexion in flight or to the dispersed force direction—most likely forward.

The synthesis of findings suggests that children at this developmental stage are not yet capable of rapidly integrating upper and lower body movements into a unified, efficient motor pattern and cannot fully convert energy into increased power. This is consistent with findings by Koren et al. [25], who reported that intersegmental coordination mechanisms in 4-year-olds are still immature. The longer flight time likely reflects forward movement rather than vertical height. We attribute this to the “artificial” nature of the CMJ movement—a structured, externally guided task intended to standardize measurements. For preschoolers, however, this is a novel movement pattern that they have not yet internalized. This hypothesis is supported by the power, RSI, contact time, pace, and verticality parameters, none of which confirmed the biomechanical effectiveness of the arm swing. Preschool children lack established synergies between the upper and lower body. As coordination of arms and legs is still developing, arm swing is not optimally synchronized with take-off, resulting in dispersed energy and a longer execution time. This lack of synchronization may prevent the potential biomechanical benefits of arm swing (e.g., greater force and a higher RSI) from being fully realized. Gieysztor et al. [26] similarly found that movement synchronization is essential for the functional benefit of arm swing in children aged 5–7.

These developmental biomechanical differences can be explained by gender- and sex-specific components of motor development. In this age range, girls generally show higher levels of coordination and balance, while boys display greater muscular strength. This is consistent with findings by Koren et al. [25], who found that better coordination significantly contributes to jump height in preschool children. Similarly, Gadzic in Milanov [35] found significantly greater strength in boys (age 6), while Navarro-Patón et al. [36] observed better coordination and balance in girls (age 5).

## 5. Conclusions

The findings of our study confirm that investigating the biomechanical characteristics of movement patterns in the preschool period requires adjustments to measurement protocols, which differ significantly from those used with adults. Our results highlight the need to develop protocols that go beyond data from force plates and similar devices and include three-dimensional kinematic analysis using 3D cameras. This represents a limitation of the present study, as the measurement systems employed do not allow for comprehensive three-dimensional kinematic capture, potentially constraining the detailed analysis of movement quality. Implementing such an approach would provide a more complete understanding of jump dynamics, including the flight phase, and allow for a more precise evaluation of movement quality and coordination.

To further validate these findings, it would be appropriate to expand research to other fundamental movement patterns, such as running and climbing, and to examine the developmental dynamics of individual patterns across various stages of growth. Such a comprehensive approach would enable a deeper understanding of the ontogenetic development of motor skills and support educators, parents, sports teachers, and other professionals in planning and implementing developmentally supportive activities in the preschool period.

## Figures and Tables

**Table 1:** Changes in CMJ performance without and with arm swing in preschool children.

Sex	Measure	CMJ Without Arm Swing	CMJ with Arm Swing	∆ (%)	ES	t-Value/Z-Value	*p*-Value
Boys	Contact time (s)	1.38 (0.87)	1.75 (1.20)	26.8	0.43	−4.437	<0.001
	Flight time (s)	0.30 (0.05)	0.32 (0.05)	6.7	0.40	−8.155	<0.001
	Height (cm)	11.3 (3.80)	13.0 (4.30)	15.0	0.45	−8.452	<0.001
	Power (W/kg)	9.5 (3.10)	9.3 (2.80)	−2.1	0.07	1.102	0.272
	Pace (steps/s) *	0.74 (0.52–1.31)	0.58 (0.44–0.80)	−21.6	0.41	−5.653	<0.001
	RSI (m/s) *	0.10 (0.05–0.19)	0.08 (0.05–0.12)	−0.20	0.36	−4.714	<0.001
	Verticality *	2.50 (1.27–5.00)	1.85 (0.97–3.81)	−0.26	0.07	−1.500	0.134
Girls	Contact time (s)	1.36 (1.02)	1.64 (0.87)	20.6	0.30	−3.766	<0.001
	Flight time (s)	0.30 (0.05)	0.31 (0.06)	3.3	0.20	−6.873	<0.001
	Height (cm)	11.2 (3.60)	12.6 (4.40)	12.5	0.39	−7.411	<0.001
	Power (W/kg)	9.5 (3.50)	9.1 (2.70)	4.2	0.13	2.063	0.040
	Pace (steps/s) *	0.67 (0.53–1.02)	0.60 (0.48–0.82)	−10.4	0.11	−3.602	<0.001
	RSI (m/s) *	0.09 (0.06–0.17)	0.08 (0.05–0.11)	−11.1	0.37	−3.951	<0.001
	Verticality *	2.29 (1.28–4.13)	1.85 (0.88–3.70)	−19.2	0.06	−1.397	0.162

Note: CMJ parameters significantly increased or decreased with arm swing, with *p* < 0.05 indicating significance. Arm swing primarily enhanced jump height, contact time, and flight time while slightly reducing pace, the RSI, and verticality. * denotes using the median and interquartile range (25th–75th percentiles).

## Data Availability

The original contributions of the data analyzed in this study are included in this article. Inquiries regarding these data or the raw data can be directed to vilko.petric@ufri.uniri.hr or sanja.ljubicic@ufri.uniri.hr (corresponding author).

## Abbreviations

The following abbreviations are used in this manuscript:

CMJ	Countermovement jump without arm swing
CMJA	Countermovement jump with arm swing
RSI	Reactive strength index

## References

[1] True L., Pfeiffer K.A., Dowda M., Williams H.G., Brown W.H., O’Neill J.R., Pate R.R. (2017). Motor Competence and Characteristics within the Preschool Environment. J. Sci. Med. Sport.

[2] Ortega F.B., Cadenas-Sánchez C., Sánchez-Delgado G., Mora-González J., Martínez-Téllez B., Artero E.G., Castro-Piñero J., Labayen I., Chillón P., Löf M. (2015). Systematic Review and Proposal of a Field-Based Physical Fitness-Test Battery in Preschool Children: The PREFIT Battery. Sports Med..

[3] Payne V.G., Isaacs L.D. (2017). Human Motor Development: A Lifespan Approach.

[4] Zhao P., Ji Z., Wen R., Li J., Liang X., Jiang G. (2021). Biomechanical Characteristics of Vertical Jumping of Preschool Children in China Based on Motion Capture and Simulation Modeling. Sensors.

[5] Petrić V., Ljubičić S., Novak D. (2025). Normative Data for Vertical Jump Tests in Pre-School Children Aged 3 to 6 Years. Biomechanics.

[6] Petrić V. (2021). Osnove Kineziološke Edukacije.

[7] Lundgren S.S., Nilsson J.Å., Ringsberg K.A., Karlsson M.K. (2011). Normative Data for Tests of Neuromuscular Performance and DXA-derived Lean Body Mass and Fat Mass in Pre-pubertal Children. Acta Paediatr..

[8] Seefeldt V. (1984). Physical Fitness in Preschool and Elementary School-Aged Children. J. Phys. Educ. Recreat. Dance.

[9] Bobbert M.F., Casius L.J.R. (2005). Is the Effect of a Countermovement on Jump Height Due to Active State Development?. Med. Sci. Sports Exerc..

[10] Ebben W.P., Fauth M.L., Garceau L.R., Petushek E.J. (2011). Kinetic Quantification of Plyometric Exercise Intensity. J. Strength Cond. Res..

[11] Lees A., Vanrenterghem J., De Clercq D. (2004). Understanding How an Arm Swing Enhances Performance in the Vertical Jump. J. Biomech..

[12] Harman E.A., Rosenstein M.T., Frykman P., Rosenstein R.M., Kraemer W.J. (1991). Estimation of Human Power Output from Vertical Jump. J. Strength Cond. Res..

[13] Taylor M.J.D., Cohen D., Voss C., Sandercock G.R.H. (2010). Vertical Jumping and Leg Power Normative Data for English School Children Aged 10-15 Years. J. Sports Sci..

[14] Ramírez-Vélez R., Correa-Bautista J.E., Lobelo F., Cadore E.L., Alonso-Martinez A.M., Izquierdo M. (2017). Vertical Jump and Leg Power Normative Data for Colombian Schoolchildren Aged 9-17.9 Years: The FUPRECOL Study. J. Strength Cond. Res..

[15] Walters G.W.M., Cooper S., Carlevaro F., Magno F., Boat R., Vagnetti R., D’Anna C., Musella G., Magistro D. (2025). Normative Percentile Values for the TGMD-3 for Italian Children Aged 3–11 + Years. J. Sci. Med. Sport.

[16] Harsted S., Hestbæk L., Holsgaard-Larsen A., Hein Lauridsen H. (2024). Dynamic Lower Limb Alignment During Jumping in Preschool Children: Normative Profiles and Sex Differences. J. Mot. Learn. Dev..

[17] Payne V.G., Isaacs L.D. (2020). Introduction to Motor Development. Human Motor Development.

[18] Raffalt P.C., Alkjaer T., Simonsen E.B. (2017). Intra-Subject Variability in Muscle Activity and Co-Contraction during Jumps and Landings in Children and Adults. Scand. J. Med. Sci. Sports.

[19] Ljubičić S., Antekolović L., Petrić V. (2022). Integration of Bilateral Coordination in Children’s Motor Learning Process. Rev. Za Elem. Izobr..

[20] Glatthorn J.F., Gouge S., Nussbaumer S., Stauffacher S., Impellizzeri F.M., Maffiuletti N.A. (2011). Validity and Reliability of Optojump Photoelectric Cells for Estimating Vertical Jump Height. J. Strength Cond. Res..

[21] Bosco C., Luhtanen P., Komi P.V. (1983). A Simple Method for Measurement of Mechanical Power in Jumping. Eur. J. Appl. Physiol..

[22] Bogataj Š., Pajek M., Hadžić V., Andrašić S., Padulo J., Trajković N. (2020). Validity, Reliability, and Usefulness of My Jump 2 App for Measuring Vertical Jump in Primary School Children. Int. J. Environ. Res. Public Health.

[23] Healy R., Kenny I.C., Harrison A.J. (2016). Assessing Reactive Strength Measures in Jumping and Hopping Using the Optojump^TM^ System. J. Hum. Kinet..

[24] Hopkins W.G., Schabort E.J., Hawley J.A. (2001). Reliability of Power in Physical Performance Tests. Sports Med. Auckl. NZ.

[25] Koren K., Pišot R., Šimunič B. (2017). Vertical Jump Height in Young Children—A Longitudinal Study in 4- to 6-Year Old Children. Ann. Kinesiol..

[26] Gieysztor E., Dawidziak A., Kowal M., Paprocka-Borowicz M. (2024). Jumping Motor Skills in Typically Developing Preschool Children Assessed Using a Battery of Tests. Sensors.

[27] Anghel M. (2019). The Influence of Sensory Systems in Motor Development to the Preschool Child. Ser. IX Sci. Hum. Kinet..

[28] Wang J.-L., Sun S.-H., Lin H.-C. (2022). Relationship of Quantitative Measures of Jumping Performance with Gross Motor Development in Typically Developed Preschool Children. Int. J. Environ. Res. Public Health.

[29] Gillen Z.M., Shoemaker M.E., McKay B.D., Bohannon N.A., Gibson S.M., Cramer J.T. (2022). Influences of the Stretch-Shortening Cycle and Arm Swing on Vertical Jump Performance in Children and Adolescents. J. Strength Cond. Res..

[30] Mosier E.M., Fry A.C., Lane M.T. (2019). Kinetic Contributions of The Upper Limbs During Counter-Movement Verical Jumps With and Without Arm Swing. J. Strength Cond. Res..

[31] Pleša J., Kozinc Ž., Smajla D., Šarabon N. (2022). The Association between Reactive Strength Index and Reactive Strength Index Modified with Approach Jump Performance. PLoS ONE.

[32] Anicic Z., Janicijevic D., Knezevic O.M., Garcia-Ramos A., Petrovic M.R., Cabarkapa D., Mirkov D.M. (2023). Assessment of Countermovement Jump: What Should We Report?. Life.

[33] Mizuguchi S., Sands W.A., Wassinger C.A., Lamont H.S., Stone M.H. (2015). A New Approach to Determining Net Impulse and Identification of Its Characteristics in Countermovement Jumping: Reliability and Validity. Sports Biomech..

[34] Eythorsdottir I., Gløersen Ø., Rice H., Werkhausen A., Ettema G., Mentzoni F., Solberg P., Lindberg K., Paulsen G. (2024). The Battle of the Equations: A Systematic Review of Jump Height Calculations Using Force Platforms. Sports Med..

[35] Gazdic A., Milanov A. (2021). Differences in Motor Abilities between Preschool Boys and Girls. Sport Sci..

[36] Navarro-Patón R., Lago-Ballesteros J., Arufe-Giráldez V., Sanmiguel-Rodríguez A., Lago-Fuentes C., Mecías-Calvo M. (2021). Gender Differences on Motor Competence in 5-Year-Old Preschool Children Regarding Relative Age. Int. J. Environ. Res. Public Health.

